# Dynamic Adsorption Characteristics of Cr(VI) in Red-Mud Leachate onto a Red Clay Anti-Seepage Layer

**DOI:** 10.3390/toxics10100606

**Published:** 2022-10-12

**Authors:** Yibo Zhang, Yue Yu, Hao Qin, Daoping Peng, Xing Chen

**Affiliations:** 1 School of Emergency Management, Xihua University, Chengdu 610039, China; 2 Faculty of Geosciences and Environmental Engineering, Southwest Jiaotong University, Chengdu 611756, China

**Keywords:** clay anti-seepage layer, red-mud leachate, Cr(VI), Visual MINTEQ simulation, dynamic adsorption

## Abstract

Red-mud leachate from tailings ponds contains Cr(VI), which can pollute groundwater via infiltration through anti-seepage layers. This paper investigates leachate from a red-mud tailings pond in southwest China and the red clay in the surrounding area to simulate the adsorption of Cr(VI) onto clay at different pHs, using geochemical equilibrium software (Visual MINTEQ). We also performed dynamic adsorption testing of Cr(VI) on a clay anti-seepage layer. The dynamic adsorption behaviors and patterns in the dynamic column were predicted using the Thomas and Yoon–Nelson models. Visual MINTEQ predicted that Cr(VI) adsorption in red-mud leachate onto clay was 69.91%, increasing gradually with pH, i.e., adsorption increased under alkaline conditions. Cr(VI) concentration in the effluent was measured using the permeability test through a flexible permeameter when the adsorption saturation time reached 146 days. At a low seepage rate, Cr(VI) adsorption onto the clay anti-seepage layer took longer. Saturation adsorption capacity, *q*_0_, and adsorption rate constant, *K_th_*, were determined using the Thomas model; the Yoon–Nelson model was used to determine when the effluent Cr(VI) concentration reached 50% of the initial concentration. The results provide parameters for the design and pollution prediction of the clay anti-seepage layer of red-mud tailings ponds.

## 1. Introduction

Red mud is a mud-like industrial solid waste with very fine particles produced during alumina production in bauxite mines, which features strong alkalinity, high salt content, and heavy-metal elements. At present, it is mainly disposed of in tailings ponds [1,2], wherein red mud mixes with water produced during smelting and rainwater to produce red-mud leachate. Due to the abundant free bases, such as NaOH, NaHCO_3_, and NaAl(OH)_4_, in red mud, when it is dissolved in liquid, a large number of free basic anions are produced to form a strongly alkaline, highly concentrated industrial wastewater containing heavy metals, such as arsenic and chromium, as well as other pollutants, such as fluorides [3,4,5]. When artificial anti-seepage layers (such as geotextile and high-density polyethylene (HDPE) membranes) of the tailing’s ponds are broken or fail, red-mud leachate directly seeps into the clay anti-seepage layer and, after completely penetrating the clay layer, pollutes the groundwater [6,7]. In addition, the strong alkalinity and high ionic strength of the red-mud leachate causes salinization of the clay anti-seepage layer, changing its structure and chemical composition, thus impacting its strength and causing concerns about the safety and stability of the tailings pond [5,8]. The heavy-metal component Cr(VI) of red-mud leachate entering the environment can cause serious soil and groundwater contamination, and long-term exposure leads to systemic poisoning symptoms as well as mutagenic and carcinogenic effects [9,10,11]. Therefore, it is critical to study the dynamic adsorption characteristics of Cr(VI) in red-mud leachate on clay anti-seepage layers and improve the design scheme of the clay layer on the basis of understanding the heavy-metal adsorption patterns.

Currently, the treatment of heavy metals in polluted water bodies primarily focuses on adsorption and ion exchange [12,13,14]. The adsorption method is more effective in treating Cr(VI) pollution, with high performance levels and low cost, and is highly regarded by researchers [15,16,17]. Many studies have been conducted on Cr adsorption, mostly focusing on its chemical behavior, migration patterns in saturated soils, and the impact of various factors on heavy-metal adsorption, such as heavy metal species, soil type, initial concentration, mixing intensity, and adsorption time [18,19]. Zhang et al. [20] investigated Cr(VI) migration through soil using dynamic adsorption experiments in soil columns and stated that Cr(VI) was not easily adsorbed by soil when the initial pH of the solution was high. An acidic environment was favorable for Cr adsorption and stabilization in soil, where Cr(VI) was more effectively immobilized in soil and not easily leached by low-pH wastewater. Deng et al. [21] synthesized modified kaolin clay (MKC) using ultrasound-assisted co-precipitation, and further studied Cr(VI) adsorption on MKC through soil column tests. Enhanced adsorption was confirmed by X-ray diffraction (XRD), energy dispersive X-ray spectroscopy (EDX), scanning electron microscopy (SEM), and Fourier transform infrared spectroscopy (FTIR), and it could be applied to Cr(VI) remediation in soil and groundwater. Samuel et al. [22] examined the effectiveness of cationic hydrogels for Cr(VI) removal from groundwater and soil through soil column tests and confirmed that a combination of cationic hydrogels with multi-pulse soil flushing is a relatively effective way to remove Cr(VI) from contaminated soil, achieving better adsorption. Campillo-Cora et al. [23] proposed that partitioning the solid phase and solution in soil determined the mobility of contaminants, such as heavy metals, through competitive adsorption and desorption binary experiments. Soil contains varying high levels of organic matter; in neutral soils, the pH is the most important variable affecting this. Matern et al. [24] studied the leaching characteristics of Cr(VI) in two chromite-ore-processing residue samples obtained from unlined landfills in Kanpur district, northern India, and through soil column experiments, concluded that Cr(VI) leaching was not limited by slow release kinetics. Based on the literature, many researchers used model simulations to investigate Cr(VI) adsorption capacity. For example, Khan et al. [25] adopted an artificial neural network to model Cr(VI) adsorption and investigated Cr(VI) adsorption through the acidic preparation of rice-husk charcoal in an aqueous solution, and concluded that it had a high adsorption capacity for Cr(VI) and neural network modeling effectively simulated Cr(VI) adsorption in aqueous solution. Liu et al. [26] evaluated the migration of Cr(VI) in soil using column and interval experiments and predicted the behavior of Cr(VI) in soil using the Yoon–Nelson and Thomas models. They proposed that soil texture was the key factor affecting Cr(VI) migration in soil, and Fe oxide content was the most critical factor impacting Cr(VI) adsorption onto soil. In soils with a relatively low proportion of sand and iron oxide content, Cr(VI) could cause greater damage.

At present, most research focuses on Cr(VI) adsorption capacity and its migration and transformation in soils using static experiments and soil column tests, as well as batch tests to quantitatively investigate the effect of a single factor, such as pH, solution concentration, temperature, time, and material dosing, on adsorption under set conditions and improving adsorption efficiency through modification of adsorbent materials [27,28,29]. The static adsorption test can adequately explain the characteristics of Cr(VI) adsorption mechanisms and obtain equilibrium adsorption parameters, while dynamic testing can accurately show the transport of heavy metals in soil and obtain the parameters relevant to solute transport and adsorption, closely resembling the actual situation [26,30]. In this study, we combined numerical simulation and indoor experiments, as well as Visual MINTEQ numerical simulation, soil column dynamic adsorption tests, and mathematical models, to study the dynamic adsorption characteristics of Cr(VI) in red-mud leachate in the clay anti-seepage layer in red-mud tailings ponds. The results provide a reference basis for the parameters of pollutant prediction design for the clay anti-seepage layer of red-mud tailings ponds.

## 2. Materials and Methods

### 2.1. Red-Clay Properties

The red-clay samples used in this study were taken around a red-mud tailings pond in Southwest China; their physical properties and chemical compositions are summarized in Table 1. Samples were first dried and then analyzed using X-ray fluorescence quantitative analysis (Shimadzu XRF-1800, Kyoto, Japan). Physical properties, such as water content as well as liquid and plastic limits, were measured according to the American Society for Testing and Materials (ASTM) standards [31].

The moisture content of each sample was analyzed according to ASTM D2216. Fifty grams of each red-clay sample was dried in an oven at 110 ± 5 °C for 24 h until a constant mass was reached. The moisture content was then calculated based on the mass of water and dried samples. The initial moisture content of the red clay was 13.5%. The specific gravity (Gs) of the samples was 2.71, according to ASTM D854, which is similar to that of quartz sand. Atterberg limits were measured per ASTM D4318. The liquid limit (LL) and plastic limit (PL) were 46.1 and 29.8, respectively, with a corresponding plasticity index (PI = LL − PL) of 16.3. The standard proctor test was conducted on the red-clay samples, following ASTM D698, to determine the maximum dry unit weight and optimum water content, which were 24% and 1.64 g/cm^3^, respectively. The cation exchange capacity (CEC) of the red clay was about 18.6 cmol(+)·kg^−1^. The major chemical components of red clay were SiO_2_, Al_2_O_3_, Fe_2_O_3_, K_2_O, and MgO. These components comprised 90.15% of the total mass of the red clay.

### 2.2. Leachate Properties

The red-mud leachate used in this study was collected from the drainage pipe of a red-mud tailings pond in Southwest China. Various parameters, including pH, electric conductivity (EC), and oxidation-reduction potential (ORP), of the leachate samples were measured immediately after sampling. The leachate sample was sealed in an HDPE sample bottle and brought back to the laboratory for testing. The chemical composition analysis was conducted following ASTM C618. Leachate samples were filtered through 0.45 µm filter paper and preserved with trace-grade nitric acid (HNO_3_). The elemental contents of aluminum, calcium, sodium, magnesium, potassium, silicon, and chromium were determined by inductively coupled plasma-optical emission spectrometry (Vista-MPX CCD Simultaneous ICP-OES, Varian Inc., Palo Alto, CA, USA) at Chengdu University of Science and Technology. Anions, including chloride, fluoride, nitrate, and sulfate, were determined using ion chromatography (Shimadzu HIC-SP, Kyoto, Japan) at Southwest Jiaotong University. The chemical composition and relevant parameters are shown in Table 2.

### 2.3. Numerical Simulation of Cr(VI) Adsorption onto Red Clay

Geochemical modeling was used in this study to simulate the effect of clay on Cr(VI) adsorption at different pH values. The numerical model Visual MINTEQ (ver. 3.1) developed by USEPA was used to identify the predominant oxidation states [32]. The Visual MINTEQ3.1 model was theoretically founded according to the soil chemical equilibrium theory established by Professor W. L. Lindsay [33]. The model divides chemical substances in aqueous solution into components and species, and species are formed through chemical reactions between components. Therefore, each chemical substance can be represented by components, and “n” chemical substances can be represented by “m” basic components, with equilibrium constants of reactions included in the Visual MINTEQ 3.1 database. The chemical parameters and ion concentrations during chromium adsorption onto clay was simulated using Visual MINTEQ, as shown in Table 2. A two-step modeling process was conducted with MINTEQ, which was described in detail in our previous studies [34,35].

### 2.4. Dynamic Adsorption Test of Red-Mud Leachate onto a Clay Anti-Seepage Layer

**Figure 1:**
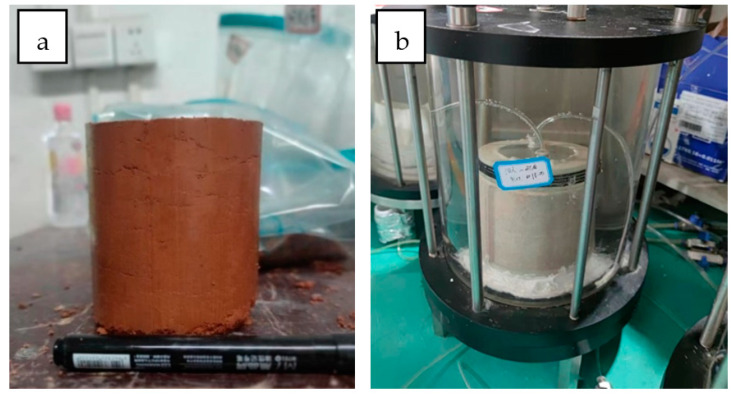
(**a**) Test sample preparation and (**b**) dynamic adsorption test setup.

The test setup is shown in Figure 1. The clay samples were sieved through 5 mm and dried in a drying oven at 105–110 °C for 12 h. The clay samples were sprayed with the required amount of water by a sprayer at the optimum moisture content, stirred thoroughly and saturated in a plastic bag for 48 h. The clay samples saturated at the optimum moisture content were poured into a light compaction cylinder in three layers and compacted in layers of 25 blows each. After compaction, the compacted clay was removed from the compaction cylinder with a soil extractor. The height of the compacted clay was 10 cm and the half diameter was 5 cm, which was the soil sample for this dynamic adsorption test (Figure 1a). The dynamic adsorption test was carried out using a flexible wall permeameter (Figure 1b). During penetration, the effective confining pressure was fixed at 24.0 kPa, and the water head of red-mud leachate influent was 160 cm to simulate the real permeability at the bottom of the red-mud tailings pond. The details of red-mud leachate used in the dynamic experiments is shown in Table 2. The Cr(VI) concentration in the effluent was measured at different time intervals.

## 3. Results and Discussion

### 3.1. Static Adsorption Simulation

Visual MINTEQ 3.1 was used to simulate the adsorption characteristics of Cr(VI) onto clay in red-mud leachate at pH values of 4, 6, 8, 10, and 12, respectively. The percentage of chromium content in each form of chromium compounds and the adsorption capacity (*Ac*) were calculated as follows:

(1) $$W=\frac{C_{i}}{C_{t}}$$

(2) $$Ac=W_{t1}-W_{t2}$$

where *W* is the percentage of chromium content, *C_i_* is the concentration of each form of chromium compounds, *C_t_* is the concentration of total chromium; adsorption capacity (*Ac*) refers to the difference in the total mass percentage of chromium before (*W_t_*_1_) and after (*W_t_*_2_) adsorption.

**Figure 2:**
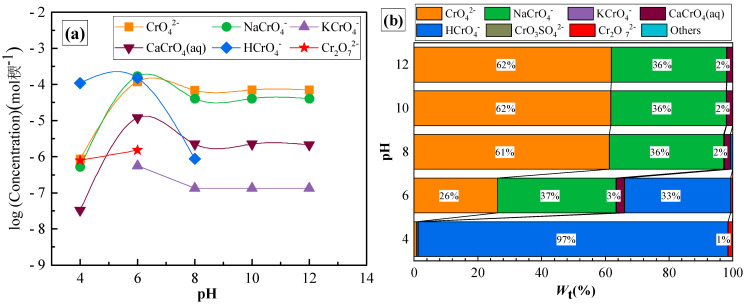
(**a**) Variations in chromium concentration with pH before adsorption and (**b**) fractional concentrations of various forms of total chromium at different pHs before adsorption.

Figure 2 shows the variations in fractional concentrations of different Cr forms in the leachate prior to adsorption following the change in pH. The form of Cr(VI) in solution is pH-dependent. When the pH was between 4 and 6, the binding ability of H^+^ to CrO_4_^2−^ was stronger than Ca^2+^, and Cr(VI) largely existed in the form of HCrO_4_^−^. The fractional concentrations of the main forms of Cr(VI) were in the following order: HCrO_4_^−^ > CrO_4_^2−^ > CaCrO_4_ (aq). When the pH was between 6 and 7.5, CrO_4_^2−^ > HCrO_4_^−^ > CaCrO_4_ (aq). When the pH was between 8 and 12, the fractional concentrations of the main forms of Cr(VI) were: CrO_4_^2−^ > NaCrO_4_^−^ > CaCrO_4_ (aq) due to the decrease in H^+^ concentration. The concentration of HCrO_4_^−^ gradually decreased with the increase in pH and, finally, approached 0. When the pH was > 8, CrO_4_^2−^, NaCrO_4_^−^, and CaCrO_4_ (aq) changed relatively little, stabilizing at 62, 35, and 1%, respectively. A similar trend of Cr(VI) forms varying with changes in pH was observed in the simulation of Cr(VI) forms in the groundwater of a chromium mine [36].

**Figure 3:**
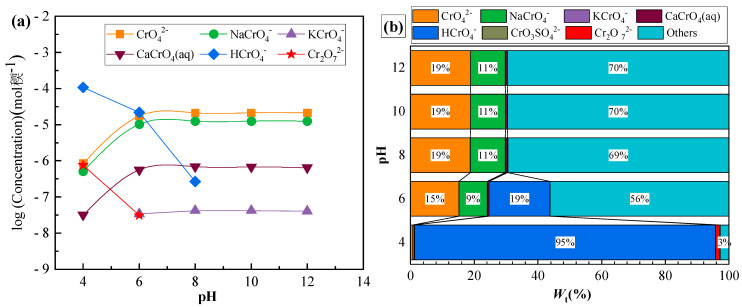
(**a**) Variations in chromium concentration with pH after adsorption; (**b**) fractional concentrations of various forms of total chromium at different pHs after adsorption.

Figure 3 displays the changes in fractional concentrations of different forms of chromium in the leachate after adsorption with variations in pH. It clearly shows that the chromium concentration decreased significantly with increasing pH, especially for CrO_4_^2−^ and NaCrO_4_^−^, which decreased remarkably by approximately 20 and 40%, respectively. The adsorption efficiency of Cr(VI) was substantially impacted by pH. The adsorption effect of clay on HCrO_4_^−^ under strong acid conditions was not significant. When the pH increased and the solution turned alkaline, adsorption gradually increased and, finally, leveled off. Electrostatic repulsion occurred between the anionic Cr(VI) and the clay particles because the Cr(VI) forms changed, which led to an increase in negative charge, resulting in decreased adsorption efficiency [37].

**Figure 4:**
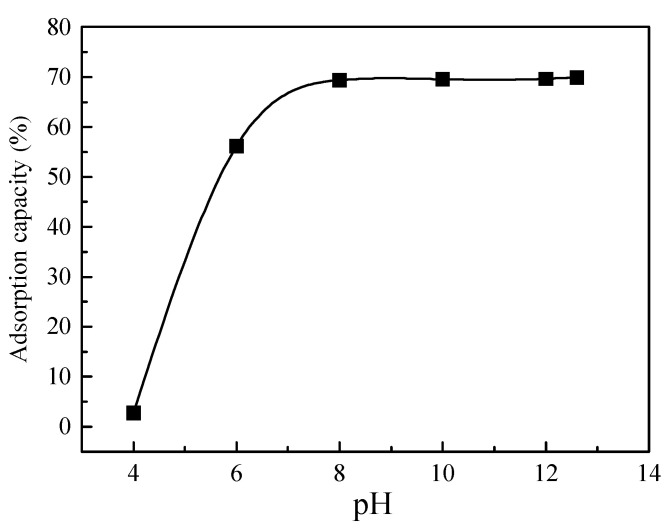
Adsorption capacity of chromium at different pH values.

Figure 4 shows Cr(VI) adsorption onto clay at various pH values. With an increase in pH, Cr(VI) adsorption gradually enhanced, and the adsorption isotherm gradually stabilized after pH entered the alkaline range and finally leveled off, indicating saturation [38]. Under acidic conditions, Cr(VI) mainly exists as HCrO_4_^−^ in the soil, and the electrostatic repulsion between HCrO_4_^−^ and the clay surface is significantly lower than that between Cr_2_O_7_^2−^ and the clay surface; Cr(VI) is primarily adsorbed onto the clay surface as HCrO_4_^−^ through the coordination reaction [39]. Under alkaline conditions, Cr(VI) adsorption onto clay tends to level off, and the functional group Si-OH on the clay surface undergoes a deprotonation reaction: Si-OH = Si-O + H^+^; thus, their abundance is significantly reduced. Additionally, OH^−^ is generated through the reaction Si-OH + CrO_4_^2−^ = Si-CrO_4_^−^ + OH^−^, which competes with CrO_4_^2−^ in solution for adsorption [40]. When the pH is >7, the negative charge in the soil solution increases due to the change in Cr forms, and the electrostatic repulsion between the solution and the negative charges on the clay surface grows, both of which lead to decreased adsorption rate. Tang et al. [41] found similar results in a study on the removal performance of Cr(VI) in a constructed rapid infiltration system, where Cr(VI) adsorption onto clay gradually increased with increasing pH in the solution; adsorption was improved under alkaline conditions.

As shown in Figure 4, clay had a good adsorption capacity for Cr(VI) in red-mud leachate under alkaline conditions. Therefore, a clay anti-seepage layer can effectively block the infiltration of Cr(VI) through adsorption during permeation, thus reducing Cr(VI) pollution from red-mud leachate into the surrounding environment or groundwater [42].

Table 3 shows the Visual MINTEQ 3.1 simulation of Cr(VI) concentrations in the leachate before and after adsorption onto clay. Cr(VI) mainly existed in the forms of CrO_4_^2−^ and NaCrO4^−^ in the leachate (pH = 12), accounting for 62.5 and 35.7% of the total chromium, respectively, and a small amount of Cr(VI) was in the forms of KCrO_4_^−^ and CaCrO_4_ (aq). After adsorption, CrO_4_^2−^ and NaCrO_4_^−^ accounted for 18.8 and 10.7% of the total chromium in the leachate, respectively. The clay adsorbed 69.9% of Cr(VI) in the red-mud leachate, showing reasonable adsorption. Since clay had a good adsorption capacity for Cr(VI) in red-mud leachate under alkaline conditions and the pH of red-mud leachate was 12, the pH of the clay anti-seepage layer for Cr(VI) adsorption in red-mud leachate was set to 12.

### 3.2. Dynamic Adsorption Test Results

In dynamic adsorption, breakthrough curves are commonly used to describe the adsorption of the contaminants in solution by the absorbent [43]. The prediction of the breakthrough curve for the clay anti-seepage layer effluent is essential for the anti-seepage design of red-mud tailings ponds.

**Figure 5:**
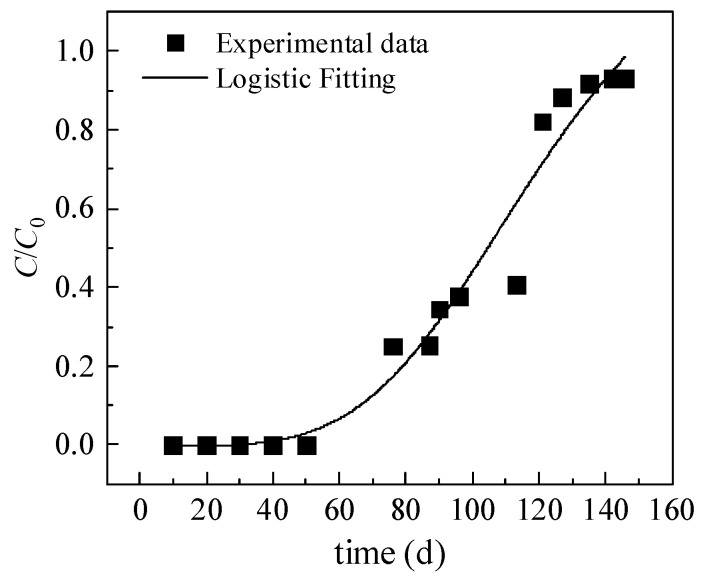
Dynamic adsorption test results with logistic fitting curve.

Figure 5 shows the results of the dynamic adsorption test on Cr(VI). The breakthrough point was set at the moment when the C/C_0_ level reached 0.05 (where C is Cr(VI) concentration in the effluent exiting the liner and C_0_ is the initial Cr(VI) concentration in the leachate). Adsorption was considered to have reached equilibrium when the effluent concentration reached >90% of the initial concentration. As Figure 5 indicates, C/C_0_ was 0 in the initial stage because the clay anti-seepage layer had not reached hydraulic equilibrium and there was no leachate effluent at that time. As the test proceeded, a small amount of leachate seeped through and exited from the bottom of the clay layer; however, the small amount of leachate effluent and the strong adsorption capacity of clay at the initial stage did not change the C/C_0_ level. As adsorption continued, at 60 d, Cr(VI) in the red-mud leachate began to break through the clay layer; at 105 d, the Cr(VI) concentration in the leachate effluent exiting the bottom of the clay layer reached 50% of the initial Cr(VI) concentration; at 121 d, the effluent Cr(VI) concentration increased to 82% of the initial concentration. Between 60–121 d, C/C_0_ increased relatively rapidly, indicating that the clay layer gradually reached hydraulic equilibrium and the soil became saturated. The adsorption efficiency of the clay was higher at this stage; however, as adsorption proceeded, Cr(VI) adsorption gradually decreased, leading to a relatively quick increase in the C/C_0_ value. The Cr(VI) concentration in the effluent reached 93% of the initial concentration at 146 d, and the adsorption gradually equilibrated. According to the Groundwater Quality Standard of China (GB/T14848-2017), the maximum permissible Cr(VI) concentration in class III groundwater is 0.05 mg/L. Regarding the effectiveness of the clay anti-seepage layer, when C/C_0_ > 0.03, the effluent Cr(VI) concentration penetrating through the liner exceeded the maximum permissible concentration and the clay layer failed.

The adsorption curve of the dynamic adsorption process can be divided into three segments, i.e., the adsorption, adsorption mass transfer, and adsorption saturation zones [44,45,46,47]. The speed of red-mud leachate movement through the clay layer had a large influence on Cr(VI) adsorption rate. In the initial stage, adsorption occurred largely on the surface. Due to the high plasticity index and large specific surface area of bentonite, the adsorption rate was high at this stage; as it proceeded, the negative charge of the solution increased, the electrostatic repulsion between the solution and the negative charge on the clay surface grew, and Cr(VI) adsorption declined. As the adsorption continued, the binding sites on the clay surface gradually became saturated, and Cr(VI) adsorption primarily took place on the internal binding sites of the clay. Cr(VI) migration to the interior of the clay had to overcome the capillary pressure from the pores, and, therefore, the adsorption rate gradually decreased [48]. According to the principle of adsorption and mass transfer, when the seepage velocity of the leachate was low, the mass transfer resistance into the clay surface increased, hindering the contaminants from penetrating the clay layer [49]. As the flow rate of red-mud leachate was very low during the test, the migration rate of red-mud leachate in the clay anti-seepage layer was slow, resulting in a longer adsorption time. Therefore, it took longer for Cr(VI) to completely break through the clay layer [50].

The breakthrough curve data were fitted using a logistic function, as follows:

(3) $$y=\frac{A_{1}-A_{2}}{1+{\left(\frac{t}{t_{0}}\right)}^{p}}+A_{2}$$

where *y* is the value of *C*/*C*_0_; *t* is the test time; and *p*, *A*_1_, *A*_2_, and *t*_0_ are the empirical parameters obtained through the fitting using this mathematical function.

Table 4 shows the fitting parameters for the mathematical model. It indicates that the fitting results of the logistic function and the actual dynamic adsorption test data match, with an R^2^ of 0.96. The logistic function can adequately fit the dynamic adsorption of Cr(VI) onto the clay layer, thereby producing reliable test data.

### 3.3. Dynamic Models

Indoor soil column adsorption tests obtain corresponding parameters through simple mathematical models, which are then simply applied to real site conditions [51,52]. Since Cr(VI) in red-mud leachate penetrated the clay layer at a slow pace, it took a long time for Cr(VI) to completely break through the clay layer. Hence, mathematical models were used to predict the adsorption behavior and patterns within the dynamic soil column. Many mathematical models can be used to analyze adsorption column test results, such as the Bohart–Adams, Bed Depth Service Time, Clark, Wolborska, Thomas, and Yoon–Nelson models, among which the Thomas model has been extensively applied [53,54,55,56,57,58]. The Thomas model is usually adopted to describe the dynamic characteristics of the adsorption column and calculate the saturated adsorption capacity and adsorption rate constant, which are, therefore, applicable to any adsorption system [59]. The Yoon–Nelson model is a semi-empirical model that does not consider the adsorption flow rate or adsorbent dosage in fitting [60]. It is simple, only requiring a few known parameters, and the values obtained can be used for comparison of the adsorption rates. Therefore, the Thomas and the Yoon–Nelson models were used here to describe the column adsorption process.

The theoretical maximum adsorption capacity and the adsorption rate constant can be obtained using the Thomas model, via the following equation:

(4) $$\frac{C_{t}}{C_{0}}=\frac{1}{1+\exp\left[\left(\frac{k_{Th}}{Q}\right)\left(q_{0}M-C_{0}V\right)\right]}$$

where *k_Th_* is the Thomas rate constant in L/(mg/d); *q*_0_ is the maximum adsorption capacity in mg/g; *M* is the filling mass of the soil column, measured at 317.45 g; *V* is the effluent volume in mL; *Q* is the flow rate at 1.129 × 10^−3^ L/d; *C*_0_ is the Cr(VI) concentration before adsorption in mg/L; and *C_t_* is the Cr(VI) concentration of the effluent at time *t* in mg/L.

Taking logarithms on both sides to transform the Thomas model into linear form:

(5) $$\ln\left(\frac{C_{0}}{C}-1\right)=\frac{k_{th}Q_{0}M}{Q}-k_{Th}C_{0}t$$

The Yoon–Nelson model is expressed as follows:

(6) $$\frac{C_{t}}{C_{0}}=\frac{1}{1+\exp\left[k\left(\tau -t\right)\right]}$$

where *k* is the rate constant in h^−1^; and *τ* is the time at which the Cr(VI) concentration of leachate effluent is 1/2 of the initial Cr(VI) concentration.

**Figure 6:**
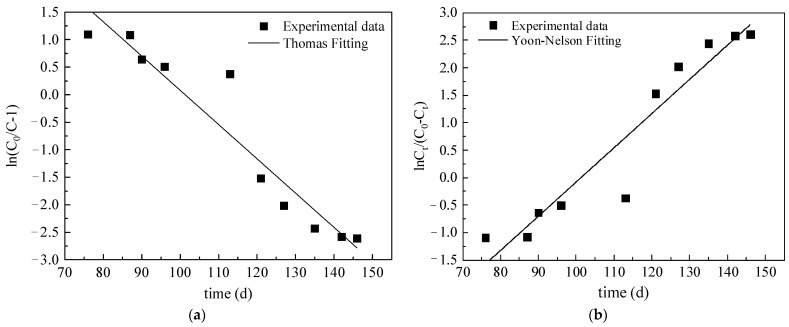
Fitting diagrams of (**a**) the Thomas and (**b**) Yoon–Nelson models.

Transforming the Yoon–Nelson model into linear form:

(7) $$\ln\left(\frac{C_{t}}{C_{0}-C_{t}}\right)=Kt-\tau K$$

a linear graph was plotted according to the linear relationship of *t*, as the x coordinates, and ln(*C*_0_/*C*-1) as well as ln[*C_t_*/(*C*_0_ − *C_t_*)] as the y coordinates, as shown in Figure 6. The linear forms of the two models were used to analyze the raw data, with specific parameters shown in Table 5. Table 5 shows that the coefficients of determination, R^2^, obtained by fitting the experimental data were uniformly >0.9, indicating that both the Thomas and the Yoon–Nelson models adequately fitted the experimental data. The maximum adsorption capacity, *q*_0_, of the clay layer for Cr(VI) in red-mud leachate was calculated using the Thomas model, which was approximately 0.634 mg/kg^−1^. Its adsorption rate constant was relatively low. The Yoon–Nelson model was adopted to calculate *τ*, the time at which *C*/*C*_0_ = 50%. The result was 101 d, which was closer to the experimental result of 105 d, further indicating that the Yoon–Nelson model could be effectively applied to this adsorption process.

Studies have shown that the faster the flow rate into the solution, the earlier the penetration time as well as the time to reach adsorption saturation, while an increase in the initial solution concentration also shortens the whole process of adsorption saturation appropriately [51,61]. The adsorption rate increased with the decrease in the flow rate, and the lower flow rate had higher adsorption efficiency under the same initial concentration. Zhang et al. [20] studied the adsorption and desorption of Cr(VI) in alkaline soil and found that the initial concentration and flow rate did not have a significant effect on the desorption process, and the desorption process of Cr(VI) in alkaline soil was accompanied by an obvious tailing phenomenon. The adsorption penetration curves can be calculated and analyzed by using Thomas model and Yoon–Nelson model, and the time *τ* for the effluent concentration to reach 50% of the initial concentration is obtained by using Yoon–Nelson model, which is similar to the results of this study.

## 4. Conclusions

We used geochemical equilibrium software (Visual MINTEQ) simulation, dynamic adsorption experiments in soil columns, and mathematical models to analyze the adsorption behavior and patterns and to investigate the dynamic adsorption characteristics of Cr(VI) in red-mud leachate onto a clay anti-seepage layer. We drew the following conclusions.

(1) The geochemical equilibrium software (Visual MINTEQ) simulation indicated that Cr(VI) adsorption in red-mud leachate onto clay was 69.9%, which was considered adequate. With increasing pH in the solution, Cr(VI) adsorption gradually increased, thereby achieving better adsorption under alkaline conditions.
(2) The soil column breakthrough test revealed that the Cr(VI) concentration in the effluent reached 93% of the initial concentration when the adsorption time reached 146 d. The experimental adsorption data were fitted with a logistic function, so as to describe the variations in concentration ratio over time. The logistic breakthrough model properly fit the dynamic adsorption process. The adsorption of Cr(VI) onto the clay anti-seepage layer under low flow-rate conditions was more persistent, requiring a longer time for Cr(VI) to completely penetrate the clay layer.
(3) Both the Thomas and the Yoon–Nelson models showed good correlations between their fitting results and the experimental measurements. The maximum adsorption capacity, *q*_0_, of the clay layer for Cr(VI) in red-mud leachate was approximately 0.634 mg/kg^−1^, according to the Thomas model. The Yoon–Nelson model estimated the time to reach the breakthrough point with *C*/*C*_0_ = 50% at 101 d, similar to the experimental result of 105 d. The parameters obtained from both models were reliable and could be used to predict the breakthrough curve of Cr(VI) in a clay layer.

## Figures and Tables

**Table 1:** Physical properties and chemical composition of red clay.

**Physical Properties**								
Liquid limit (%)	Plastic limit (%)	Plasticity index (%)	Initial moisture content (%)	Optimum water content (%)	Maximum dry density (g/cm^3^)	Specific gravity (Gs)	Compression index (*Cc*)	Swelling index (*Cs*)
46.1	29.8	16.3	13.55	24	1.64	2.71	0.05	0.024
**Chemical composition**								
Constituent	SiO_2_	Al_2_O_3_	Fe_2_O_3_	CaO	Na_2_O	MgO	K_2_O	Other
Weight (%)	55.96	26.16	6.37	0.06	0.12	0.66	1.0	9.66

**Table 2:** Chemical properties of red-mud leachate.

pH	EC@25 °C (mS-cm^−1^)	ORP (mV)	Ionic Strength (mM)	Elemental Composition (mg·L^−1^)											
				Al	Ca	Na	Mg	K	Si	Cl^−^	F^−^	NO_3_^−^	SO_4_^2^^−^	Cr	Cr(VI)
12.6	51.1	−110.0	484.3	745.5	57.1	10,650.0	10.5	81.8	89.9	6490.5	121.8	483.4	7453.3	5.9	1.76

**Table 3:** Speciation distribution of Cr(VI) forms in the leachate before and after adsorption.

**Before Adsorption**			
**Form**	**Molar Concentration (mol** **·L^−1^)**	**Mass Fraction (%)**	**Activity (mol** **·L^−1^)**
CrO_4_^2−^	7.09 × 10^−5^	62.514	2.02 × 10^−5^
NaCrO_4_^−^	4.05 × 10^−5^	35.658	2.96 × 10^−5^
KCrO_4_^−^	1.33 × 10^−7^	0.117	9.7 × 10^−8^
CaCrO_4_ (aq)	1.94 × 10^−6^	1.17	2.15 × 10^−6^
**After Adsorption**			
**Form**	**Molar Concentration (mol** **·L^−1^)**	**Mass Fraction (%)**	**Activity (mol** **·L^−1^)**
CrO_4_^2−^	2.1347 × 10^−5^	18.813	6.0869 × 10^−6^
NaCrO_4_^−^	1.2177 × 10^−5^	10.731	8.8983 × 10^−6^

**Table 4:** Dynamics parameters of the model.

Test Conditions	Fitting Formula	R^2^
Cr = 1.76 mg·L^−1^ Q = 1.129 × 10^−3^ L·d^−1^	$y=\frac{-1.433}{1+{\left(t∕120.839\right)}^{4.243}}+1.429$	0.96

**Table 5:** Dynamic parameters of the models.

Fixed Parameters	Thomas Model			Yoon-Nelson Model			
	*k_Th_* (L·mg^−1^·d^−1^)	*q*_0_ (mg·kg^−1^)	R^2^	K	*τ*_mod_(d)	*τ*_exp_(d)	R^2^
M = 317.45 g Q = 1.129 × 10^−3^ L·d^−1^ Cr(VI) = 1.76 mg·L^−1^	0.0354	0.634	0.920	0.0623	101	105	0.920

## Data Availability

The data are available upon reasonable request from the corresponding author.
